# Tracheotomy

**Published:** 1851-07

**Authors:** 


### TRACHEOTOMY.

This operation was successfully performed on the 3d of March in a case of *Edematous Laryngitis*, by Dr. W. D. Stephenson, of Mount Pleasant, Alabama, assisted by Dr. E. P. M. Johnson. The subject was a young mulatto woman who had been confined a fortnight previously, and from exposure had contracted a cold, which resulted in such swelling of the parts about the glottis as threatened instant suffocation. All the general and local remedies usually applied in such cases having been resorted to ineffectually, and the symptoms growing continually more urgent, tracheotomy was determined upon. At this time the patient was in indescribable agony; her features were pale and shrunken; extremities cold, nails livid, surface bathed in a cold perspiration. The relief following the operation was instantaneous; a full, deep inspiration succeeded, and the patient was asleep. For a canula, the physicians substituted a vial broken in the middle, the neck being secured in the tracheal incision. On the 12th of March, says Dr. S., breathing was partly performed in the natural way, and on closure of the orifice, it was found that the patient experienced no difficulty in inspiration. The edges of the wound were brought together by a suture and adhesive strips, and on the 20th she was well. Y.

